# The Impact of Parameter Selection and Setup Conditions on Image Quality of an On-Board Helical Kilovoltage Computed Tomography System

**DOI:** 10.7759/cureus.29244

**Published:** 2022-09-16

**Authors:** Riley C Tegtmeier, William S Ferris, John E Bayouth, Wesley S Culberson

**Affiliations:** 1 Department of Medical Physics, University of Wisconsin School of Medicine and Public Health, Madison, USA; 2 Department of Human Oncology, University of Wisconsin School of Medicine and Public Health, Madison, USA

**Keywords:** imaging parameters, on-board imaging, computed tomography, adaptive radiotherapy, igrt, image quality, helical kvct, clearrt, radixact, tomotherapy

## Abstract

Purpose

To evaluate the imaging performance of an on-board helical kilovoltage computed tomography (kVCT) system mounted on a helical tomotherapy unit for various imaging parameters and setup conditions.

Methods

Images of a commonly used computed tomography (CT) image quality phantom were acquired while varying the selection of available parameters (anatomy, mode, body size) as well as phantom positioning and size. Image quality metrics (IQM) including noise, uniformity, contrast, CT number constancy, and spatial resolution were compared for parameter and setup variations.

Results

The use of fine mode improved noise and contrast metrics by 20-30% compared to normal mode and by nearly a factor of two compared to the coarse mode for otherwise identical protocols. Uniformity, CT number constancy, and spatial resolution were also improved for fine mode. Thorax and pelvis anatomy protocols improved noise, uniformity, and contrast metrics by 10-20% compared to images acquired with head protocols. No significant differences in CT number constancy or spatial resolution were observed regardless of anatomy choice. Increasing body size (milliampere second (mAs)/rotation) improved each image quality metric. Vertical and lateral phantom shifts of up to ±6 cm degraded noise and contrast metrics by up to 30% relative to the isocenter while also worsening uniformity and CT number constancy. IQM were also degraded substantially with the use of annuli to increase the phantom diameter (32 cm vs. 20 cm). Despite variations in image characteristics among the investigated changes, most metrics were within manufacturer specifications when applicable.

Conclusion

This work demonstrates the dependence of image quality on parameter selection and setup conditions for a helical kVCT system utilized in image-guided and adaptive helical tomotherapy treatments. While the overall image quality is robust to variations in imaging parameters, care should be taken when selecting parameters as patient size increases or positioning moves from the isocenter to ensure adequate image quality is still achieved.

## Introduction

Modern-day, high-precision radiation therapy has led to the continued development of imaging modalities for image-guided radiation therapy (IGRT) and adaptive radiation therapy (ART) implementation. In both instances, the imaging system’s ability to provide adequate image quality to sufficiently delineate anatomic structures is necessary to avoid erroneous patient setup and treatment delivery [1,2]. Computed tomography (CT) is a noninvasive, radiological imaging technique based on X-ray transmission to generate three-dimensional cross-sectional images required for IGRT and/or ART. The quality of the final image produced with this modality is limited by several factors, including polyenergetic X-ray spectra, X-ray scatter, detection efficiency, etc. However, image data processing techniques can be introduced to moderate these effects and improve image quality.

A helical kilovoltage computed tomography (kVCT) system named ClearRT™ is available for use on the Accuray Radixact® (Sunnyvale, CA) helical tomotherapy system. The system consists of a kilovoltage (kV) X-ray source and a flat-panel detector (FPD) mounted orthogonal to the megavoltage (MV) treatment beam. Clinically, it can be utilized to verify patient setup and positioning in IGRT and to adaptively perform contour refinement and dose reconstruction.

One potential limitation in using an FPD (due to material composition) is artifacts introduced by image lag, which refers to residual signals present in image frames subsequent to the frame in which the signal was generated [3]. Multiple detector effects (array lag, scintillator afterglow, etc.) may lead to a temporal delay between x-ray incidence and signal readout, resulting in characteristic arc-shaped artifacts and other, more subtle artifacts. Lag correction techniques can be implemented to improve image uniformity for rotationally asymmetric and off-centered objects. Additionally, scatter signals collected by detector elements can degrade image quality by increasing noise and reducing contrast. Scatter correction and noise reduction techniques are often introduced to mitigate these effects.

Image reconstruction for this helical kVCT system is performed with a Hilbert-transform-based filtered back-projection (FBP) reconstruction algorithm. The updated reconstruction software (Version 3.0.1.0), released in early 2022, utilizes various correction techniques, including noise reduction and scatter and lag correction, to improve overall image quality. While the methods behind these techniques are briefly discussed, the goal of this study was to evaluate the impact of parameter selection and setup conditions on image quality and provide the first in-depth analysis of the impact of the updated reconstruction software. 

## Materials and methods

For this system, the user cannot directly adjust the tube technique (kilovoltage peak (kVp) and milliampere second (mAs)); rather, scan acquisition parameters are specified by the selection of specific protocols named according to their expected use. The anatomy parameter determines the tube potential and reconstructed slice interval (Head-100 kVp, 1.2 mm reconstructed slice interval, Thorax-120 kVp, 1.8 mm, Pelvis-140 kVp, 1.8 mm), while body size adjusts beam fluence. The field-of-view (FOV) determines the filtration type and the detector-collimator offset in the plane of rotation. Lastly, the mode defines the longitudinal beam width, helical pitch, and views-per-rotation (Fine, Normal, or Coarse). Changes to this parameter have been observed to most impact image quality as variation in the beam width influences the scatter signal while variation in views-per-rotation impacts output (mAs/rotation) and patient dose [4, 5]. For additional information on system and protocol specifications, the reader is referred to the manufacturer's guide [6].

Conventional noise reduction techniques to correct scatter operate on radiation data comprised of both a primary and a scatter component. The purpose of this correction is to estimate the scatter component and filter this from the data. However, applying this correction to the entire data set to optimize noise reduction for the high-noise scatter component often over-corrects for and degrades the resolution of the low-noise primary component. The noise reduction approach utilized by the updated software operates only on the scatter component. In theory, this achieves sufficient noise reduction with minimal resolution degradation (due to "smoothing") as the components of the data are optimized independently.

In short, the lag correction approach for this system utilizes "dark" frames (the x-ray source is turned off) as anchors for the model. Per request by the manufacturer, a detailed discussion of this specific technique is reserved for a later date. While this approach generally improves uniformity for off-centered objects, it may introduce intermittent, light streaking artifacts most sensitive to high-contrast objects.

The Radixact system available at this institution is released for research purposes only, and thus, in this study, a Catphan®-504 (The Phantom Laboratory, Inc., Greenwich, NY) phantom was used to evaluate image quality. For information on phantom specifications, the reader is referred to the manual provided by the manufacturer [7]. In total, fifty scans were evaluated. Imaging parameters and setup conditions, including mode, body size, anatomy, and phantom size and positioning, were varied to determine the impact of this variation on overall image quality. Notably, the scan mode and phantom size were varied to alter the scatter component of the detected signal to evaluate the implications of the noise reduction approach, while phantom positioning was varied to assess the implications of the lag correction approach. A field of view of 440 mm with an aluminum bowtie filter was used regardless of the additional parameter selections to ensure identical filtration and detector-collimator offset across all comparisons. For each acquisition, image quality metrics (IQM) including noise, uniformity, contrast, CT number constancy, and spatial resolution were evaluated as outlined by Tegtmeier et al. [5]. The noise was defined as the standard deviation of pixel values within some homogenous region of interest (ROI) and was reported as a percentage of the mean pixel value within this ROI [8]. Image uniformity was assessed with the uniformity index (UI) metric, which is defined as the difference between the mean Hounsfield units (HU) in the periphery and the center ROIs of some homogenous phantom module [9]. Low-contrast visibility (LCV) [10], referring to the ability to distinguish between materials with similar attenuation properties, was calculated as:

$LCV = 2.75\times \frac{\sigma _{PV,Poly}+\sigma _{PV,LDPE}}{\mu _{PV,Poly}-\mu _{PV,LDPE}}$

The contrast-to-noise ratio (CNR) [9] for the Delrin insert was calculated as:

$CNR = \left | \frac{\mu _{HU,Insert}-\mu _{HU,BG}}{\sqrt{\sigma _{Insert}^{2}+\sigma _{BG}^{2}}} \right |$

Analysis was primarily performed with ImageJ software (National Institutes of Health, U.S.), while spatial resolution in the axial plane was evaluated with Python code.

## Results

Noise

**Figure 1:**
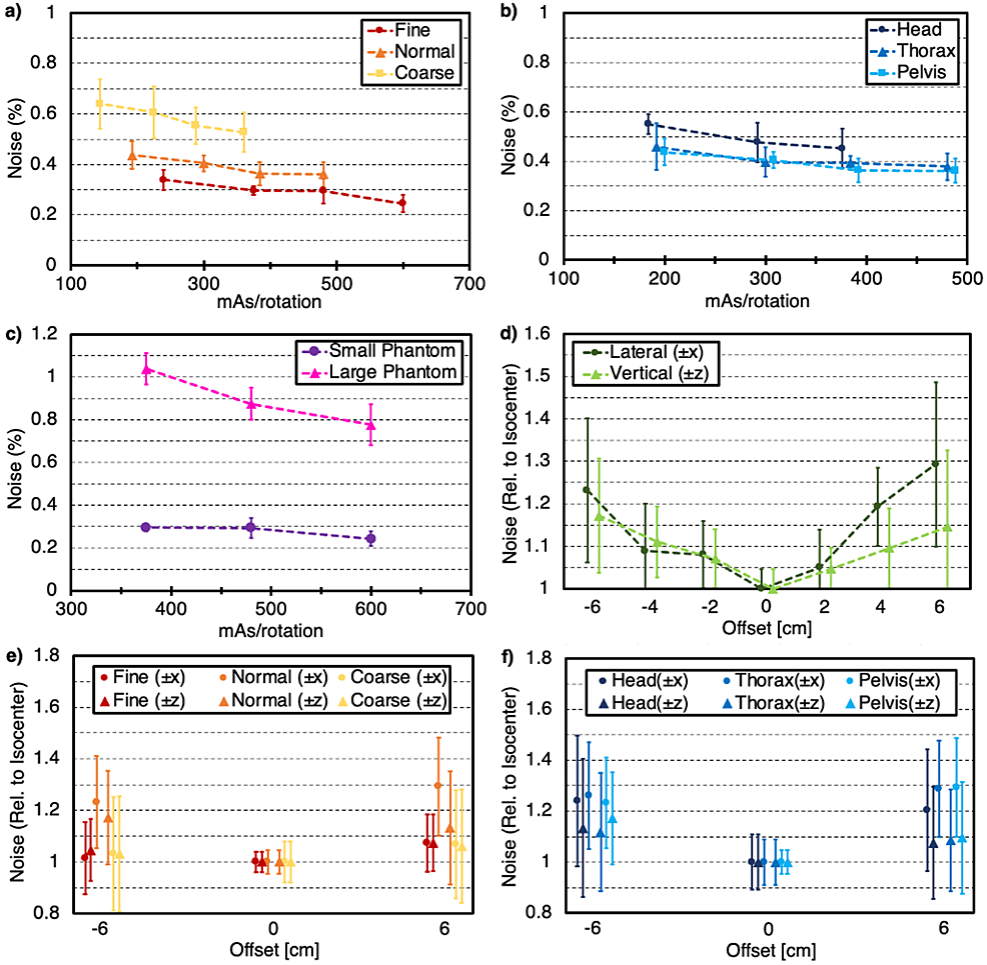
Noise as a percentage of the mean pixel value for various parameter selection and setup conditions (*a*) as a function of mAs/rotation for each mode, (*b*) as a function of mAs/rotation for each anatomy, (*c*) as a function of mAs/rotation for small (20 cm) and large (32 cm) phantom sizes, (*d*) as a function of phantom position for both lateral and vertical shifts up to ± 6 cm, (*e*) as a function of phantom position for the maximum lateral and vertical shifts (± 6 cm) for each mode, (*f*) as a function of phantom position for the maximum lateral and vertical shifts (± 6 cm) for each anatomy. Error bars represent one standard deviation from the mean value for each individual acquisition.

Figure 1a shows noise as a function of body size (mAs/rotation) for each mode for scans acquired with the pelvic anatomy. The application of Fine mode (50 mm beam width at isocenter) reduced noise by ~25% compared to Normal mode (100 mm) and a factor of ~2 compared to Coarse mode (~140 mm) for similar values of mAs/rotation. It is important to note that the application of Fine mode increases standardized dose output by up to a factor of two for otherwise identical scan parameters compared to Coarse mode based on computed tomography dose index (CTDIvol) values reported on the console. However, because mAs/rotation roughly scales with dose, comparing metrics for similar mAs/rotation values implies comparing metrics for similar dose values as well. Figure 1b depicts the noise as a function of mAs/rotation for each anatomical group for scans acquired in Normal mode. The use of Thorax anatomy showed similar results to those for the Pelvis anatomy, while the use of the Head anatomy increased noise by roughly 20%. Standardized dose output according to reported CTDIvol values was roughly constant regardless of anatomy chosen for otherwise identical parameters. Note that for Figures 1a-1b, data points from left to right for each mode or anatomy indicate small, medium, large, and X-large body size selections (Head anatomy does not include the X-large selection). Noise as a function of mAs/rotation for the phantom with and without annuli for scans acquired with Fine mode and pelvic anatomy is shown in Figure 1c. Increasing the phantom diameter from 20 cm (small) to 32 cm (large) increased noise by a factor of 3-3.5. However, for these acquisitions, the noise was still within ~1%. Noise for the large phantom was also more dependent on mAs/rotation. For these scans, small body size was not included.

Furthermore, the phantom was moved both laterally (x-axis) and vertically (z-axis) from the isocenter in 2 cm offsets (up to 6 cm) in both positive and negative directions to evaluate the lag correction approach. When looking into the bore, the +x-axis was defined as "right" while the -x-axis was defined as "left". Likewise, the +z-axis was defined as "up" while the -z-axis was defined as "down". Figure 1d shows noise as a function of phantom positioning for both lateral and vertical shifts for scans acquired in the Normal mode, Pelvis anatomy, and large body size. These values are reported relative to the noise for the phantom at the isocenter. For lateral offsets, the noise increased by up to ~30% relative to the scans at the isocenter for the largest shifts (±6 cm) while noise increased by less than 20% for vertical offsets of the same magnitude. Additionally, the mode was varied to determine how relative noise values compared to the largest shifts both laterally and vertically, as observed in Figure 1e. These scans were acquired with Pelvis anatomy and large body size parameter selections. Noise relative to the isocenter was higher for the Normal mode than for the Fine and Coarse modes for vertical and lateral shifts of ±6 cm. Lastly, relative noise values for the largest shifts for each anatomical selection are observed in Figure 1f for scans acquired with Normal mode and Large body size. A similar increase in noise when shifting the phantom ±6 cm laterally and vertically is observed regardless of anatomy selection. The noise increased significantly more for lateral shifts (~25-30%) than for vertical shifts (~8-15%). Note that while data was only acquired at discrete integer offsets of 0 cm, ±2 cm, ±4 cm, and ±6 cm, the data points in Figures 1d-1f were offset on the plot to improve readability.

Uniformity

**Figure 2:**
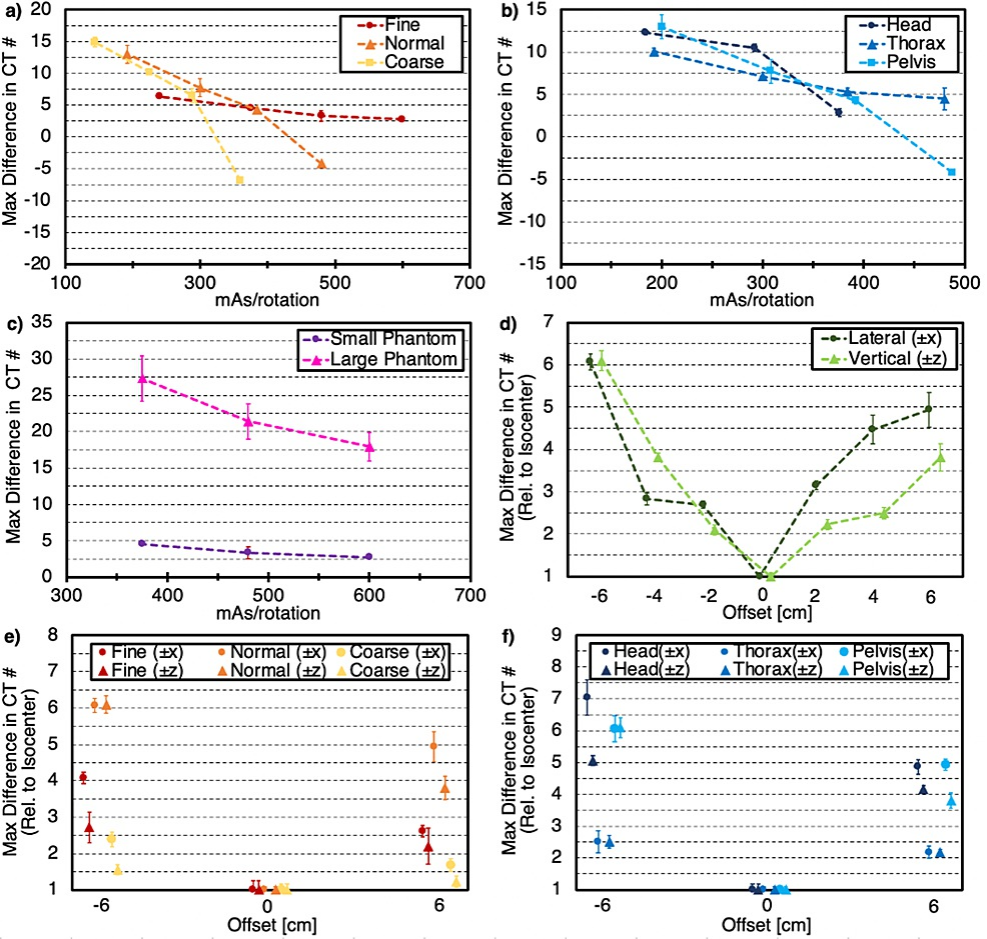
Uniformity index (UI) for various parameter selection and setup conditions (*a*) as a function of mAs/rotation for each mode, (*b*) as a function of mAs/rotation for each anatomy, (*c*) as a function of mAs/rotation for small (20 cm) and large (32 cm) phantom sizes, (*d*) as a function of phantom position for both lateral and vertical shifts up to ± 6 cm, (*e*) as a function of phantom position for the maximum lateral and vertical shifts (± 6 cm) for each mode, (*f*) as a function of phantom position for the maximum lateral and vertical shifts (± 6 cm) for each anatomy. Error bars represent one standard deviation from the mean value for each individual acquisition.

For uniformity analysis and comparison of uniformity index (UI) values (and all additional metrics), image parameter selection and setup conditions are as described above in Figures 1a-1f. Variation in UI values was lower with the use of Fine mode, as indicated in Figure 2a. UI values for a variation in anatomy are shown in Figure 2b. The use of Thorax anatomy provided less variability in UI values as a function of mAs/rotation. Changing phantom size increased UI values by a factor of ~5-7 as shown in Figure 2c, indicating a noticeable degradation in image uniformity as phantom size is increased. When varying phantom position as seen in Figure 2d, values for UI increased by up to a factor of six at an offset of -6 cm and by up to a factor of five for +6 cm shifts in both the lateral and vertical directions. Additionally, variation in mode showed that UI was less dependent on phantom position for Fine and Coarse modes when compared to Normal mode, as shown in Figure 1e. Likewise, UI was less dependent on phantom position for Thorax anatomy when compared to Head and Pelvis anatomies (Figure 1f).

Contrast

**Figure 3:**
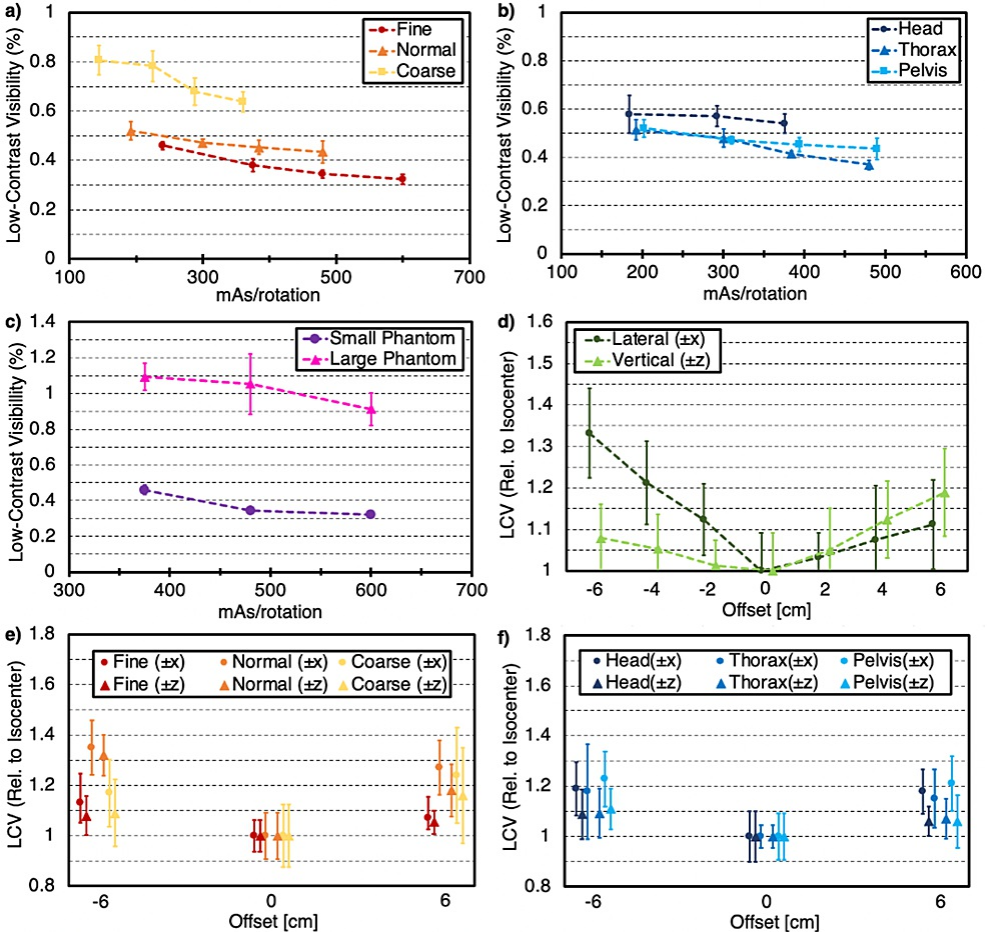
Low-contrast visibility (LCV) for various parameter selection and setup conditions (*a*) as a function of mAs/rotation for each mode, (*b*) as a function of mAs/rotation for each anatomy, (*c*) as a function of mAs/rotation for small (20 cm) and large (32 cm) phantom sizes, (*d*) as a function of phantom position for both lateral and vertical shifts up to ± 6 cm, (*e*) as a function of phantom position for the maximum lateral and vertical shifts (± 6 cm) for each mode, (*f*) as a function of phantom position for the maximum lateral and vertical shifts (± 6 cm) for each anatomy. Error bars represent one standard deviation from the mean value for each individual acquisition.

**Figure 4:**
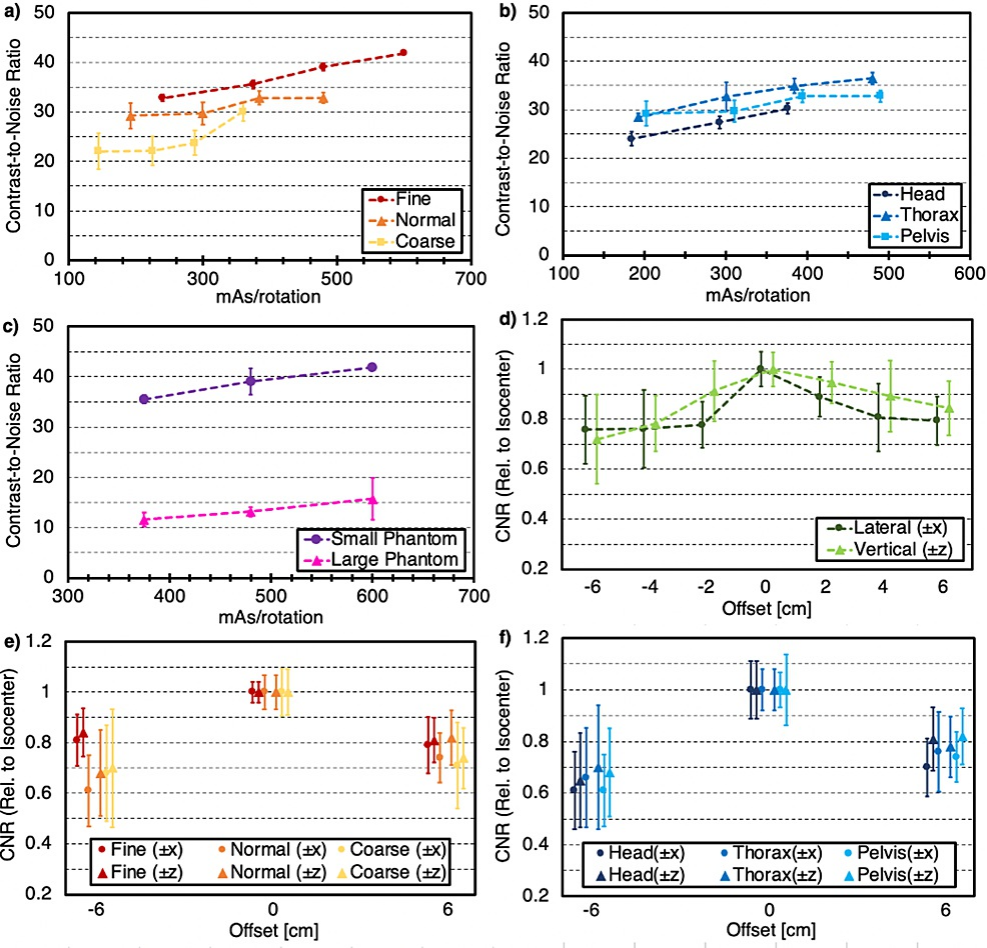
Contrast-to-noise ratio (CNR) for various parameter selection and setup conditions (*a*) as a function of mAs/rotation for each mode, (*b*) as a function of mAs/rotation for each anatomy, (*c*) as a function of mAs/rotation for small (20 cm) and large (32 cm) phantom sizes, (*d*) as a function of phantom position for both lateral and vertical shifts up to ± 6 cm, (*e*) as a function of phantom position for the maximum lateral and vertical shifts (± 6 cm) for each mode, (*f*) as a function of phantom position for the maximum lateral and vertical shifts (± 6 cm) for each anatomy. Error bars represent one standard deviation from the mean value for each individual acquisition.

Contrast performance was assessed with both the low contrast visibility (LCV) and contrast-to-noise (CNR) metrics. Use of Fine mode improved LCV and CNR by roughly 10% and 10-15%, respectively, when compared to Normal mode and by roughly 50-70% and up to 30%, respectively, when compared to Coarse mode for similar values of mAs/rotation as shown in Figures 3a and 4a, respectively. Contrast metrics were also slightly improved with the application of the Thorax anatomy when compared to the Pelvis anatomy and improved by 15-20% when compared to the Head anatomy acquisitions as seen in Figures 3b and 4b, respectively. Additionally, contrast metrics for the large phantom were degraded by roughly a factor of 2.5-3 for identical values of mAs/rotation when compared to the small phantom, as shown in Figures 3c-4c, respectively.

For lateral offsets, LCV was degraded by nearly 35% when shifting in the negative x-direction and by less than 15% when shifting in the positive x-direction. As shown in Figure 3d, values for vertical shifts showed variability in LCV of less than 10% in the negative z-direction and nearly 20% in the positive z-direction. As shown in Figure 4d, offsets in the negative direction degraded CNR metrics by over 30% for both axes, while offsets in the positive direction degraded CNR by roughly 20-25%. The variation in mode suggested that LCV and CNR relative to the isocenter were overall less dependent on phantom position for Fine mode when compared to Normal and Coarse modes, as seen in Figures 3e-4e, respectively. Lastly, similar degradation in contrast metrics was observed regardless of anatomy selection as shown in Figures 3f-4f. Additionally, lateral shifts appeared to degrade contrast slightly more than vertical shifts of the same magnitude.

CT number constancy

**Figure 5:**
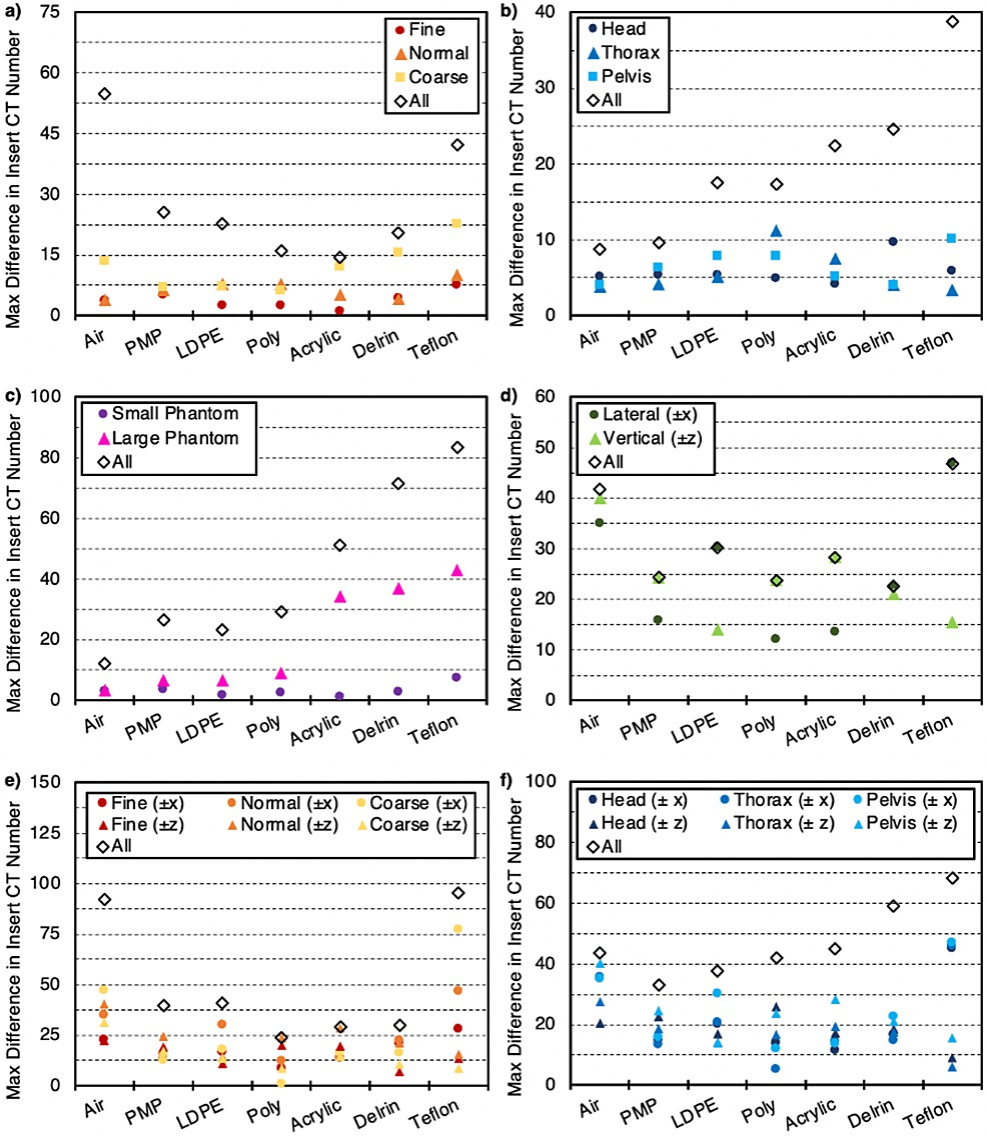
Computed tomography (CT) number constancy as the maximum difference in insert CT number for various parameter selection and setup conditions (*a*) as a function of mAs/rotation for each mode, (*b*) as a function of mAs/rotation for each anatomy, (*c*) as a function of mAs/rotation for small (20 cm) and large (32 cm) phantom sizes, (*d*) as a function of phantom position for both lateral and vertical shifts up to ± 6 cm, (*e*) as a function of phantom position for the maximum lateral and vertical shifts (± 6 cm) for each mode, (*f*) as a function of phantom position for the maximum lateral and vertical shifts (± 6 cm) for each anatomy. Note: PMP refers to polymethylpentene and LDPE refers to low-density polyethylene.

Mean CT numbers for each of the phantom inserts were quantified to determine the constancy of CT number measurement under different imaging parameters. Differences in mean CT numbers across acquisitions for all body size selections were smallest with the use of Fine mode, as values for each insert were within ~8 HU when varying from 240 mAs/rotation to 600 mAs/rotation as shown in Figure 5a. Values for Normal mode were all within ~10 HU, while differences for Coarse mode were up to ~25 HU for the high-density Teflon insert. Furthermore, variation in mean CT number across all acquisitions shown on a given plot is shown, regardless of mode or body size selections (i.e., the values producing this maximum variation could have differed in mode and/or body size). The overall differences generally increased as insert density both increased or decreased relative to water and were as large as 50 HU for the "air" insert. Figure 5b shows differences for each anatomical selection as body size was varied. Overall, differences did not show a noticeable trend based on the selection of anatomy. Maximum differences in each insert across all acquisitions regardless of anatomy or body size increased as insert density increased and reached nearly 40 HU for the high-density Teflon insert. Likewise, differences for the large phantom increased as insert density increased, and differences across all acquisitions regardless of phantom size or body size selection were up to greater than 80 HU for Teflon as seen in Figure 5c.

Differences in the mean CT number for each insert as a function of phantom position (up to ±6 cm in each direction) did not produce a noticeable trend as observed in Figure 5d. The variation in mean CT number for each mode as a function of body size and phantom position (±6 cm in each direction) is shown in Figure 5e. A similar trend was observed in Figure 5a, though the magnitude of the variation was increased due to variation in phantom position as well. Figure 5f depicts the variation in mean CT number for each anatomy as a function of body size and phantom position. Once again, a similar trend to that described in Figure 5b was observed, though the magnitude of the differences once again increased due to the variation in phantom position.

Spatial resolution

**Figure 6:**
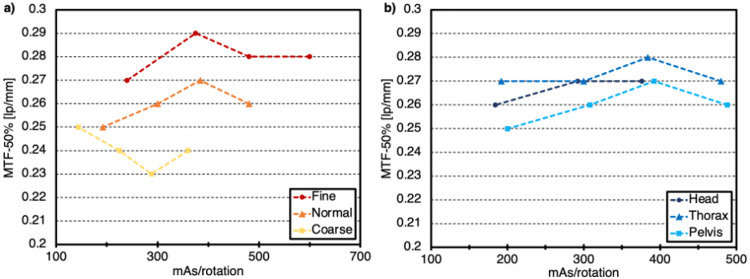
Modulation transfer function (MTF) at 50% of the original contrast value (*a*) as a function of mAs/rotation for each mode, (*b*) as a function of mAs/rotation for each anatomy.

Spatial resolution in the axial plane was measured with the high contrast resolution module of the phantom. Python code utilizing the Pylinac library was developed to derive the modulation transfer function (MTF) describing contrast recovery as a function of the spatial frequency. The MTF value at 50% of the original contrast value (MTF50%) was reported in units of line pairs per millimeter (lp/mm). Spatial resolution was only measured for scans at the isocenter and without annuli. The use of Fine mode improved spatial resolution by less than 10% relative to Normal mode and by up to 20% compared to Coarse mode for similar values of mAs/rotation as shown in Figure 6a. Overall, variation in anatomy showed little impact on the spatial resolution as all values were within 8% (Figure 6b).

## Discussion

The noise reduction, scatter, and lag correction approaches for this system were refined by the manufacturer for the latest reconstruction software version commercially available in early spring 2022. The goal of this study was to provide the first in-depth analysis of the impact of parameter selection and phantom size and positioning on IQM for this system and software. The authors would like to emphasize that these results are representative of the system available for use at this institution and are not intended to provide absolute values for these metrics. Additionally, no patient scans were performed in this research according to the current status of this system at this institution, and thus the values provided are representative of a standard image quality phantom. Nonetheless, this work provides insight into the relative image quality that can be expected as imaging parameters are varied for this system. In general, the use of Fine mode improved each IQM for similar values of mAs/rotation. Notably, Fine mode also increased scan times for the phantom length of 20 cm by nearly a factor of two compared to Coarse mode, though these times were still on the order of seconds (25 seconds for Coarse mode, 45 seconds for Fine mode). The use of Thorax anatomy slightly improved contrast metrics when compared to the higher-energy Pelvis anatomy protocols. For all other metrics, values for the Thorax and Pelvis protocols were similar. Overall, lateral shifts degraded IQM more so than vertical shifts of the same magnitude, and this trend was better observed when varying anatomy compared to mode. Additionally, image quality degradation was generally larger for shifts in the negative direction for both lateral and vertical offsets. Image degradation due to phantom shifts with a diagnostic CT scanner has been previously reported in the literature [11]. In this previous study, it was determined that noise in a single axial slice for the diagnostic scanner could vary by a factor of 2 due to off-centering of up to ±10 cm.

As is evident in the data of this study, image noise can be reduced by increasing mAs. This reduction in noise generally improves contrast metrics important for the delineation of anatomic structures. However, increasing mAs also increases the dose to the patient proportionally. Thus, noise reduction is an important way to optimize the output of CT scans by allowing for the reduction of dose while maintaining similar or improved image quality. The denoising approach used by this system operates only on the scatter component of the signal, reducing the potential to degrade image quality for already high-quality, low-noise images as well as preserving spatial resolution. Nearly all scans in this study had noise levels of less than 1% regardless of parameter selection or imaging conditions (the large phantom, low mAs/rotation acquisition had a noise level of 1.04%).

Correction of the image lag is also important to mitigate possible image degradation due to limitations in the physics of FPDs as previously discussed. In most instances of diagnostic CT, positioning the patient at the isocenter is important for several reasons. When utilizing bowtie filters (as is the case for the 440 mm FOV used across all acquisitions in the study), it is assumed that the part of the patient with the highest attenuation is aligned with the isocenter. Moving the patient from the isocenter results in a diminished ability of the bowtie filter to compensate for patient attenuation, resulting in higher or lower doses than needed for adequate image quality depending on the region of the patient in question [11]. Additionally, off-centering can result in a large variation in the signal-to-noise ratio (SNR) that propagates through the image reconstruction chain and results in non-uniform noise. Off-centering may occur under a variety of circumstances, most notably for centering of the tumor to the isocenter for treatment. Thus, it is important to introduce techniques in reconstruction algorithms for imaging systems as evaluated in this study to lessen the impact of off-centering on image quality and allow for the acquisition of adequate images under a variety of setup conditions. For images used for dose planning in adaptive treatment delivery, uniformity across the entire scan cross-section should be within the specified tolerance. According to recommendations by the American Association of Physicists in Medicine (AAPM) Task Group 66, for diagnostic-quality CT simulators, the differences in CT number between periphery and central ROIs should be within 5 Hounsfield units (HU) [12], while for MVCT systems on HT units, this difference should be within 25 HU [13]. The manufacturer's specification for this helical kVCT system is ±15 HU. Nearly all scans in this study fell within ±15 HU with the exception of scans with the large phantom and for several of the offset phantom positions. However, these tolerances are usually set with standard image quality phantoms at the isocenter, and thus this result is not necessarily unexpected.

CT number constancy is also important for dose calculation accuracy, as relative electron density information used to correct tissue inhomogeneity in the treatment planning system is derived from this data. Analysis of tolerances for HU values has suggested that variation for air/lung, soft tissue/water, and bone-equivalent materials to ensure errors in dose calculation of less than 1% should be within ±50 HU, ±20 HU, and ±50 HU, respectively, based on previous studies and guidance documents from professional bodies [14]. Thus, careful consideration should be made when determining scan protocols for images used adaptively. In general, parameters used for CT number to relative electron density calibration should match those intended to be used clinically to minimize uncertainty due to HU variation across different scan protocols [15].

Two recent publications included an analysis of image quality for this system in comparison to CT simulation and other on-board CT systems (cone-beam CT and MVCT) for several image quality metrics [4,5]. These previous studies concluded that this system performed well in comparison to the other systems for IQM important to the implementation of IGRT and ART, as the contrast was consistent with a CT simulator and overall image quality was greatly improved when compared to MVCT. However, this previous work was performed with the reconstruction software (Version 3.0.0.11), which was included with the initial system released in spring 2021. As the methods for this study were reproduced by Tegtmeier et al., a direct comparison of IQM between the updated and previous reconstruction algorithm was possible [5]. Of the 10 unique protocols previously evaluated, five were reevaluated in this study. The comparison of applicable IQM between this study and Tegtmeier et al. [5] is shown in Table 1. Based on these values, the application of the updated reconstruction software resulted in a further reduction in noise by over 20% relative to the previous software for the Thorax and Pelvis anatomy protocols and by over 30% for the head anatomy protocol. Little variation was seen in uniformity. However, it is to be expected that the software update would have little impact on uniformity for homogenous, rotationally symmetric phantoms at the isocenter. The use of the updated software further improved LCV metrics by up to ~10% for the head anatomy protocol, ~30% for Thorax protocols, and ~5% for the Pelvis anatomy protocol. Little variation was observed for CNR.

**Table 1:** Comparison of image quality metric measurements between the current study and previous study Note: FOV refers to field-of-view.

Protocol (anatomy-body size-mode-FOV)	Image quality metric							
	Noise (%)		Uniformity index		Low-contrast visibility (%)		Contrast-to-noise ratio (Delrin)	
	Update	Prev. [5]	Update	Prev. [5]	Update	Prev. [5]	Update	Prev. [5]
Head Medium Normal 440 mm	0.48	~0.70	11	~12	0.58	~0.65	27	~25
Thorax Small Normal 440 mm	0.46	~0.58	10	~7	0.51	~0.70	28	~25
Thorax Medium Normal 440 mm	0.40	~0.52	7	~7	0.48	~0.70	33	~27
Thorax Large Normal 440 mm	0.39	~0.54	5	~7	0.41	~0.53	35	~35
Pelvis Medium Normal 440 mm	0.41	~0.51	8	~6	0.48	~0.50	30	~33

Future work must be performed to assess the clinical impact of these observations on the reliability of image-guided and adaptive processes. However, the data in this study provides an initial indication that sufficient image quality as required for IGRT and ART implementation in helical tomotherapy treatments can be achieved with this system for a variety of parameter selections and imaging conditions.

## Conclusions

The impact of parameter selection and phantom positioning and size on image quality for an on-board helical kVCT system was evaluated by acquiring scans under a variety of imaging conditions. All acquisitions were reconstructed with the updated software, applying noise reduction and lag correction techniques. The use of Fine mode improved image quality overall relative to Normal and Coarse modes for otherwise identical protocols. Additionally, increasing body size (mAs/rotation) generally improved each IQM as well. However, values for reported CTDIvol were also increased by up to a factor of 2 for Fine mode or the largest body size, and thus the tradeoff between image quality and patient dose for the selection of these parameters should be considered in clinical implementation. The use of Thorax and Pelvis anatomy protocols improved image quality compared to images acquired with Head protocols. Noise, uniformity, and contrast were degraded by up to a factor of 3.5 with the use of annuli to increase the phantom diameter (32 cm vs. 20 cm), while vertical and lateral phantom shifts of up to ±6 cm degraded image quality metrics by up to over 30% relative to the isocenter. Generally, the degradation relative to the isocenter was reduced for Fine mode images. In a clinical setting, the selection of scan parameters should be based on the intended use of the image set at the discretion of the physician/physicist. Despite variation in image characteristics among the investigated changes within this study, metrics for most of the acquisitions were within the manufacturer's specifications when applicable, suggesting flexibility in the selection of parameters to provide the best tradeoff between image quality and patient dose on a case-by-case basis in clinical implementation.
